# Acquired cow’s milk sensitization after liver transplant in an adult: “clinical implications” and future strategies

**DOI:** 10.1186/s13223-019-0326-5

**Published:** 2019-02-21

**Authors:** C. Caruso, E. Pinter, E. Poli, F. Ferri, M. Merli, S. Colantuono, G. Mennini, F. Melandro, G. Rumi, R. Galandrini, S. Ginanni Corradini

**Affiliations:** 1 Allergy Unit, Presidio Columbus, Fondazione Policlinico A. Gemelli, IRCSS, Rome, Italy; 2 Dipartimento di Chirurgia Generale e Trapianti d’Organo, “Sapienza” Università di Roma, Policlinico Umberto I, Rome, Italy; 3 Clinical Immunology, Department of Clinical Medicine, Sapienza University, Rome, Italy; 4 Department of Experimental Medicine, Sapienza University of Rome, Rome, Italy; 5 Gastroenterology Unit, Department of Clinical Medicine, Sapienza University, Rome, Italy

**Keywords:** Allergy food, Transplantation, Basophils

## Abstract

**Background:**

Identifying the mechanisms responsible for the development of food allergy in liver transplant recipients is more complex as there are several different clinical scenarios related to the immunological function of the liver.

**Case presentation:**

We describe the first case of Transplant Acquired Food Allergy (TAFA) to cow milk in an adult following LT from a donor dead because of anaphylactic shock. A 67-year-old woman with primary biliary cirrhosis was referred to the Transplant Center of our hospital because of an acute-on-chronic liver failure. The donor was a 15-year-old girl deceased for anoxic encephalopathy due to food induced anaphylaxis after eating a biscuit. In the donor’s history food allergies to cow milk and eggs were present.

**Conclusion:**

This case emphasizes the need for a standardized assessment of both solid-organ donors and recipients including donor allergy history in order to detect recipients at risk for anaphylaxis due to passive IgE transfer. Despite several reports of TAFA after solid organ, especially liver, an appropriate protocol to avoid risk for the recipient doesn’t exist at the moment. The SPT (skin prick test) or specific IgE level are not enough to ensure a correct management in these cases and a correct education of the patients and the medical staff involved is absolutely necessary. It is the first case of milk allergy sensitization after solid organ transplant by passive transfer of IgE.

To the editor

In the last decades many cases of allergy to foods (particularly nuts) following solid organ transplantation, especially after liver transplant (LT), have been described [1]. If the donor does have a food allergy, different mechanisms could explain the acquired food allergy in the recipient [2]. We describe the first case of Transplant Acquired Food Allergy (TAFA) to cow milk in an adult following LT from a donor decesaed because of an anaphylactic shock. A 67-year old woman with primary biliary cirrhosis (diagnosed in 2001) was referred to the Transplant Centre of our hospital because of an acute-on-chronic liver failure. On April 2013 the patient developed jaundice and asthenia with laboratory evidence of acute-on-chronic liver failure. On admission, high levels of alanine aminotransferase and aspartate aminotransferase, total bilirubin, the international normalized ratio (INR) with a calculated Model for End-stage Liver Disease (MELD) score of 28 on a scale of 6 to 40. During hospitalization an overlap syndrome derived from the combination of primary biliary cirrhosis and autoimmune hepatitis (positivity of anti-nuclear antibodies [ANA] 1:1280 and of anti-mitochondrial antibodies [AMA] 1:1280) was diagnosed. Other causes of acute liver failure were excluded: hepatitis C antibodies (HCV Ab) and hepatitis B surface antigen (HBsAg) were negative, and urinary copper excretion, as well as transferrin saturation index were normal. After exclusion of viral and bacterial infection immunosuppressive therapy with high doses of corticosteroids was started, but as the MELD score increased to 35 the patient was evaluated for a LT. Orthotopic LT from a deceased donor was carried out 6 days after the patient’s inscription on the LT transplant waiting list. After LT the recipient started immunosuppressive therapy with mycophenolate mofetil, tacrolimus and oral prednisone as per protocol. The post-operative course was benign; after 5 days in the intensive care unit (ICU) the recipient was transferred to the ward and discharged 17 days after LT. The donor was a 15-year-old girl deceased for anoxic encephalopathy due to food-induced anaphylaxis after eating a biscuit. The donor had a history of food allergy to cow milk and eggs. During the ICU the donor was treated with intravenous high-dose corticosteroids, norepinephrine at a maximum dosage of 0.4 mcg/kg/min for 8 days and dopamine at a maximum dosage of 10 mcg/kg/min for 4 days. After the assessment of brain death, the liver and the kidneys were explanted for transplantation. The donor’s HLA phenotype was A11,26; B38,50; DR17,14; DQ2,5.

Taking into account the donor’s history, immediately after the liver transplantation an allergological work up with in vitro testing for food allergy (in particular cow milk, eggs and nuts), was performed to rule out transmission of allergies to the recipient.

The recipient did not have an atopic history including history of food allergy. During the 1st week after LT a progressive increase of eosinophils was observed on the blood count.

Blood sample collected on the post-operative day (POD) 6 showed allergic sensitization to casein (IgE 2.8 KU/L, n.v. < 0.1 KU/L) (ImmunoCAP; Thermo Fisher Scientific, Waltham, MA, USA) that were declining on day 16 (0.30 KU/L). A close follow-up of specific IgE to food allergens was performed during the following months until normalization of specific IgE levels after 7 weeks (Table 1). A Basophil Activation Test (BAT) using CD63 was carried out 1 month after LT and scored positive for casein, beta lactalbumin and egg yolk, while other food allergens scored negative [3] (Table 2).

**Table 1:** Value of specific IgE

	POD 6	POD 16	POD 47
Total IgE (< 100 kU/L)		9.22	4.60
Specific IgE			
Olive (< 0.1 kUa/L)		0.01	0.00
Cat epithelium and dander (< 0.1 kUa/L)	0.12	0.03	0.01
Egg white (< 0.1 kUa/L)		0.00	0.00
Milk (< 0.1 kUa/L)		0.25	0.03
Soyabean (< 0.1 kUa/L)	0.01	0.00	
Egg yolk (< 0.1 kUa/L)		0.02	0.01
Alpha-lactalbumin (< 0.1 kUa/L)	0.20	0.03	0.00
Beta-lactalbumin (< 0.1 kUa/L)	0.17	0.03	0.01
Casein (< 0.1 kUa/L)	2.48	0.30	0.03
Nut (< 0.1 kUa/L)		0.01	
Ovalbumin (< 0.1 kUa/L)	0.00	0.00	0.00
Ara h1 peanut (< 0.1 kUa/L)	0.01		
Ara h2peanut (< 0.1 kUa/L)	0.00		
Ara h3 peanut (< 0.1 kUa/L)	0.00		
Ara h8 PR-10 peanut (< 0.1 kUa/L)	0.02		
Ara h9 LTP peanut (< 0.1 kUa/L)	0.02		
Cor a1 PR-10 hazelnut (< 0.1 kUa/L)	0.00	0.00	0.00
Cor a8 LTP hazelnut (< 0.1 kUa/L)	0.04	0.00	
Pru p3 LTP peach (< 0.1 kUa/L)		0.01	0.01
Tri a19 0mega5 wheat (< 0.1 kUa/L)		0.00	0.00
nGal d1 Ovomucoid (< 0.1 kUa/L)	0.00	0.00	
Ara h1 peanut (< 0.1 kUa/L)	0.01		
Ara h2 peanut (< 0.1 kUa/L)	0.00		
Ara h3 peanut (< 0.1 kUa/L)	0.00		
Ara h8 PR-10 peanut (< 0.1 kUa/L)	0.02		
Ara h9 LTP peanut (< 0.1 kUa/L)	0.02		
Cor a1 PR-10 hazelnut (< 0.1 kUa/L)	0.00	0.00	0.00
Cor a8 LTP hazelnut (< 0.1 kUa/L)	0.04	0.00	
Pru p3 LTP peach (< 0.1 kUa/L)		0.01	0.01
Tri a19 0mega5 wheat (< 0.1 kUa/L)		0.00	0.00
nGal d1 Ovomucoid (< 0.1 kUa/L)	0.00	0.00	

See the positive value of casein at POD 6

**Table 2:** BAT test results

	POD 45			POD 90		
	1:100 diluition	1:1000 diluition	1:2000 diluition	1:100 diluition	1:1000 diluition	1:2000 diluition
Egg white	2.34	1.45	1.44	1.81 1.28	54.8 0.65	18.7 2.24
Egg yolk	1.44	1.56	4.86	0.48 1.13	1.6 3.5	1.14 1.76
Alpha-lactalbumin	1.54	1.16	1.81	1.86	3.23	1.92
Beta-lactalbumin	1.26	2.96	2.39	1.12	2.42	2.37
Casein	7.02	27.06	34.79	0.94	4.62	4.52
Hazelnut	1.40	2.63	1.30	1.45	2.75	1.6
Peanut	2.21	1.94	0.83	3.7	2.74	2.72
Wheat				44.15	1.16	3.71
	Negative control: 1.79			Negative control: 3.55		
	Positive control (anti-IgE): 42			Positive control (anti-IgE): 40.7		

See positive values for casein, beta lactalbumin and egg yolk at POD 45. At POD 90 egg and beta-lactalbumin scored negative; casein values significantly reduced (*p* < 0.001)

The milk- and egg-free diet was continued for 9 months, based on literature data [4] that reported a case of anaphylaxis with negative Skin Prick Testing. An epinephrine auto-injector was given to the patient in the event of accidental ingestion of those foods. Seven months after LT oral provocation tests with cow milk and hen eggs were carried out. Cow milk challenges were performed using pasteurized milk, starting with a drop of milk put on the patient’s tongue and then swallowed followed by increasing doses (0.1, 0.3, 1, 3, 10, 30, 100 mL) 20-min a part [5]. Oral food challenge with hen eggs was performed by an emulsion of both raw egg yolk and white mixed with a tolerated juice, starting with a drop and roughly doubling the doses 20-min apart until the whole egg was ingested. Both these tests were negative.

Two months after the reintroduction of milk and egg in the diet the BAT scored negative as did the skin prick tests. Basophil activation tests was performed in this patient at time two (t2) and at time 6 (months) (t6). In both measurements and for the various concentrations of allergens tested at time zero, it was negative compared to controls. None of the stimulations increased in CD63 expression compared to control patients [6].

The patient is currently on a free diet, without any clinical problem 4 years after LT.

The two kidney transplant recipients who received the organs from the same donor and were transplanted in different transplant centers, did not develop a TAFA.

Regarding solid organ transplantations several cases of TAFA, especially in paediatric recipients of LT, have been reported in the literature. The most frequent causative food allergens are peanuts, cow milk protein, eggs and soy, but the underlying physiopathological mechanisms remain incompletely understood. It has been suggested that tacrolimus, one of the most used post-LT immunosuppressive agents, might play a role in the onset of food allergy as it alters the permeability of the intestinal barrier, thus facilitating the crossing by food allergens [7].

A previous case report by Schuller et al. noted that allergic food reactions occurred after lung and liver transplantation from donors with allergic reactions but it did not occur with kidney and heart transplants from the same donors [7].

Food allergy can be passively transferred from donors to recipients of a liver transplant, and most cases described in adults occurred after deaths from food-induced anaphylaxis. In all cases reported so far, the recipient experienced a severe reaction at a variable distance from the transplantation, ranging between few days to months, until even years there have also been cases of de novo food allergy after transplantation that occurs when the donor did not have food allergy suggesting that different mechanisms of sensitization may be involved in different individuals [8].

One potential explanation for the transfer of IgE-mediated allergy in a transplant recipientis the ability of the donor’s IgE to remain viable in the hepatic sinusoids and extravascular spaces post mortem despite the normal half-life of circulating IgE is only a few days.

When the IgE-mediated allergy appears months after the transplant, donor lymphocytes in the liver allograft could be the source of IgE production in the recipient. Food allergy transfer has been well described following bone marrow transplantation where recipients were found to have donor-specific IgE producing B cells in their marrow [9].

In our patient, circulating specific IgE to casein was detectable as early as 6 days after LT, probably because of a passive transfer, but was no longer present after 3 and 6 months. Surprisingly, in our patient the BAT showed an unusual reactivity also to food other than milk. We are unable to explain this finding and may hypothesize the possible influence of the pre-existing autoimmune disorders or of the immunosuppressive therapy.

This case emphasizes the need for a standardized assessment of both solid-organ donors and recipients including donor allergy history in order to detect recipients at risk for anaphylaxis due to passive IgE transfer. Despite several reports of TAFA after solid organ, especially liver, an appropriate protocol to avoid risk for the recipient doesn’t exist at the moment. The SPT or specific IgE level are not enough to ensure a correct management in these cases and a correct education of the patients and the medical staff involved is absolutely necessary. In conclusion, this case describes the first allergic cow’s milk sensitization after solid organ transplant by passive transfer of IgE.

## Abbreviations

TAFA: Transplant Acquired Food Allergy
LT: liver transplant

## References

[1] Mitsui M, Shoda T, Natsume O, Nomura I, Narita M, Fukuda A (2017). Factors associated with development of food allergy in young children after liver transplantation: a retrospective analysis of 10 years’ experience. J Allergy Clin Immunol Pract.

[2] Newman EN, Firszt R (2018). Post-transplantation development of food allergies. Curr Allergy Asthma Rep.

[3] Santos AF, Brough HA (2017). Making the most of in vitro tests to diagnose food allergy. J Allergy Clin Immunol Pract.

[4] Vagefi PA, Blazick E, Hamilos D, Ades A, Cosimi AB, Hertl M (2009). Transference of food allergy after adult liver transplantation. Transplantation.

[5] Calvani M, Iacono ID, Giorgio V, Sopo SM, Panetta V, Tripodi S (2012). A new model for conservative food challenge in children with immunoglobulin E—mediated cow’s milk allergy. Isr Med Assoc J..

[6] Elizur A, Appel MY, Goldberg MR, Yichie T, Levy MB, Nachshon L (2016). Clinical and laboratory 2-year outcome of oral immunotherapy in patients with cow’s milk allergy. Allergy.

[7] Nowak-Wegrzyn AH, Sicherer SH, Conover-Walker MK, Wood RA (2001). Food allergy after pediatric organ transplantation with tacrolimus immunosuppression. J Allergy Clin Immunol..

[8] Schuller A, Barnig C, Matau C, Geny S, Gosselin M, Moal MC (2011). Transfer of peanut allergy following lung transplantation: a case report. Transplant Proc.

[9] Bellou A, Kanny G, Fremont S, Moneret-Vautrin DA (1997). Transfer ofatopy following bone marrow transplantation. Ann Allergy Asthma Immunol.

